# Sequencing Analysis and Identification of the Primary Peptide Component of the Dialyzable Leukocyte Extract “Transferon Oral”: The Starting Point to Understand Its Mechanism of Action

**DOI:** 10.3389/fphar.2020.569039

**Published:** 2020-10-07

**Authors:** Luis Vallejo-Castillo, Liliana Favari, Said Vázquez-Leyva, Gabriela Mellado-Sánchez, Zaira Macías-Palacios, Leonardo E. López-Juárez, Luis Valencia-Flores, Emilio Medina-Rivero, Rommel Chacón-Salinas, Lenin Pavón, Sonia Mayra Pérez-Tapia

**Affiliations:** ^1^Unidad de Desarrollo e Investigación en Bioprocesos (UDIBI), Escuela Nacional de Ciencias Biológicas, Instituto Politécnico Nacional, Mexico City, Mexico; ^2^Departamento de Farmacología, Centro de Investigación y de Estudios Avanzados del IPN, Ciudad de México, Mexico; ^3^Laboratorio Nacional para Servicios Especializados de Investigación, Desarrollo e Innovación (I + D + i) para Farmoquímicos y Biotecnológicos (LANSEIDI-FarBiotec-CONACyT), Escuela Nacional de Ciencias Biológicas, Instituto Politécnico Nacional, Mexico City, Mexico; ^4^Departamento de Inmunología, Escuela Nacional de Ciencias Biológicas, Instituto Politécnico Nacional, Mexico City, Mexico; ^5^Laboratorio de Psicoinmunología, Dirección de Investigaciones en Neurociencias, Instituto Nacional de Psiquiatría Ramón de la Fuente., Mexico City, Mexico

**Keywords:** Transferon, human dialyzable leukocyte extracts, MS sequencing, immunomodulatory drugs, oral peptides, monomeric ubiquitin

## Abstract

“Transferon Oral” is a peptide-derived product with immunomodulatory properties obtained from the lysis and dialysis of human buffy coat. Its active pharmaceutical ingredient, generically known as Dialyzable Leucocyte Extract, is a mixture of peptide populations with reproducible proportions among batches. “Transferon Oral” modulates IFN-γ, TNF-α, and IL-6 and increases the survival rate in a herpes infection murine model when oropharyngeally (ORO) administered, which correlate with clinical observations where “Transferon Oral” is used as a therapeutic auxiliary in inflammatory diseases. Notwithstanding, how a peptide-derived product elicits systemic modulation of cytokines when ORO administered remains unclear. To shed light on the pharmacology of “Transferon Oral” its peptide components must be known. Ten “Transferon Oral” batches were sequenced by mass spectrometry and the intact peptides were identified. The most abundant peptides were the monomeric human Ubiquitin (Ub), a globular low-molecular mass protein, and an Ub variant which lacks the two-terminal Gly (Ub-GG). Recombinant Ub prevented murine death when ORO administered in a herpes infection murine model. Besides, the percentage of survival increased in groups treated with Transferon Oral+Ub and decreased in groups treated with Ub-depleted “Transferon Oral” respect to the group treated with “Transferon Oral” only. Our findings indicate that the biological properties of “Transferon Oral” are partially associated to the Ub content. They suggest that Ub may activate its extracellular receptor (CXCR-4) in the stomach eliciting systemic immunomodulatory effects *via* vagus nerve. This is the first report that identifies an active component of “Transferon Oral” with the potential for the development of oral peptide immunomodulators.

## Introduction

Immunotherapy involves the stimulation, enhancement, suppression, or desensitization of the immune response to treat malignancies. Among the immunotherapeutics, stand out the human dialyzable leukocyte extracts (hDLE) which are composed of a low–molecular mass peptide mixture with immunomodulatory properties obtained from the lysis of mammalian cells. Since their first description, made in 1949 by Henry Sherwood Lawrence who also coined the term “Transfer factor” (Lawrence, 1949), dialyzable extracts have been developed in some countries such as China, Czech Republic, Cuba, the USA, and Mexico, as auxiliary drugs to treat immune system-related diseases (Ojeda et al., 2005; Zhou et al., 2013; Cardoso et al., 2017). However, the biological effects of these cellular extracts have been controversial due to the variability of the peptide content among products, which is highly dependent on their cellular origin and the manufacturing method.

In the 60s, Dr. Sergio Estrada-Parra developed and started the laboratory-scale production of an hDLE at the National Polytechnic Institute (IPN for its Spanish acronym) in Mexico, which derived into a pharmaceutical product called “Transferon Oral.” This product is obtained from lysed human buffy coats by freeze/thaw cycles and peptides with a size less than 10 kDa, the active pharmaceutic ingredient (API) of “Transferon Oral”, are separated by dialysis and ultrafiltration (Medina-Rivero et al., 2016). “Transferon Oral” has been developed as a complex drug owing to its intricate composition, i.e., its peptides components have been regarded as a unique population, and the physicochemical behavior of the peptide fraction has been determined in several batches using a large battery of analytical techniques, such as SDS-PAGE, UPLC, MS, and NMR (Crommelin et al., 2015). These analyses have evinced the high reproducibility of the peptide components of “Transferon Oral” among all batches, despite its complex composition (Medina-Rivero et al., 2014; Medina-Rivero et al., 2016; Vazquez-Leyva et al., 2019).

“Transferon Oral” has been used as an auxiliary treatment in patients with inflammatory diseases (Hernandez et al., 2013; Homber et al., 2015). “Transferon Oral” improves resolution of infectious diseases by increasing the number of systemic IFN-γ cells in ocular fungal keratitis and enhances the survival of pediatric patients with sepsis by lowering C reactive protein (CRP), rising increases total lymphocyte numbers and decreasing total neutrophil count (Santacruz-Valdes et al., 2010; Castrejon Vazquez et al., 2019). Besides, Transferon^®^, the parenteral version of “Transferon Oral” increases IFN-γ levels in serum and favors the clinical course in patients with herpes zoster infection when administered by subcutaneous route (Estrada-Parra et al., 1998).

Several models have been developed to understand the signaling pathways by which “Transferon Oral” elicits its therapeutic effect. Regarding *in vitro* models, “Transferon Oral” increases the expression of CD80/CD86 and IL-6 levels in LPS-stimulated macrophage-like THP-1 cells, whereas it induces the differentiation of IFN-γ-producing NK CD56+CD16+CD11c+ cells from CD34+ progenitor cells obtained from human umbilical cord (Ramirez-Ramirez et al., 2016; Jimenez-Uribe et al., 2019). On the other hand, “Transferon Oral” (1-25 μg/kg) reduces tumour growth and metastasis in a murine prostate cancer model, while in Herpes Simplex Virus type 1 (HSV-1) infection murine model “Transferon Oral” decreases the bloodstream levels of TNF-α and IL-6 and increases IFN-γ levels and the percent of survival when oropharyngeal administered (ORO) (Salinas-Jazmin et al., 2015; Hernandez-Esquivel et al., 2018). Nevertheless, the information provided by these models is no enough to explain how a peptide extract can modulate the immune system when administered by an enteral route. In this sense, it is essential to identify the peptide components of “Transferon Oral”, at least the most abundant, to understand the bases of the immunomodulatory properties of this blood-derived product.

The aim of this work was to the sequence of peptide components of Transferon through mass spectrometric techniques, to identify of the primary peptide component and to evaluate its relevance to the immunomodulatory properties of “Transferon Oral” using in a murine model of HSV-1 infection. Finally, we analyzed this information to propose a new hypothesis for the immunomodulatory effects of “Transferon Oral”, which will be further studied in depth later.

## Materials and Methods

### Analytical Samples

“Transferon oral,” henceforth only Transferon, was used in this study. All Transferon batches were manufactured by Pharma-FT Laboratory (Mexico City, Mexico) using a standardized method described elsewhere (Medina-Rivero et al., 2016). Briefly, human buffy coats were acquired from certified blood banks, and the cell content was disrupted by applying freeze-thaw cycles. The lysate was dialyzed through a 12-kDa membrane, and the permeate was filtrated by a 10-kDa cartridge. The obtained solution was diluted to 0.4 mg/mL using water for injection (Pisa Laboratories; Jalisco, Mexico). Transferon batches were subjected to a quality control analysis, which included sterility, identity, pH, endotoxin content, relative density, total protein content, identity, and potency. All Transferon batches were kept at -18°C until use and complied the acceptance criteria established by the manufacturer.

### MS *De Novo* Sequencing

Transferon was *de novo* sequencing to identify the proteins that provide its peptide content. Ten Transferon batches (14G18, 14G19, 15A03, 15A04, 15C10, 15D11, 15D12, 15E14, 15F17, and 15G18) were sequencing by UCDavis Genome Center (CA, USA) using standardized methods. Briefly, Transferon samples were lyophilized using a FreeZone Dry System (Labconco; MO, USA), and 2 mg of Transferon were cleaned using the ProteoExtract^®^ Protein Precipitation Kit (Calbiochem; CA, USA) according to manufacturer’s instructions. Samples were reconstituted in approximately 100 μL of 6.0 M urea (Sigma-Aldrich; SO, USA), reduced with 1-4 Dithiothreitol (Sigma-Aldrich) at 5 mM and 37°C during 30 min, and alkylated with iodoacetamide (Sigma-Aldrich) at 15 mM and room temperature during 10 min. Iodoacetamide was quenched by adding and excess of the reducing agent. Trypsin/Lys-C (Promega; WI, USA) was added in a 1:25 (enzyme:peptide) ratio an incubated at 37°C during 4 h. Then, 550 μL of 50 mM ammonium bicarbonate buffer (Sigma-Aldrich) was added and the digestion continued overnight. Samples were desalted using Macro Spin Columns (The Nest Group; MA, USA) according to the manufacturer’s instruction. Samples were recovered in a water:acetonitrile + formic acid solution (20%:80% + 0.5%) (Thermo Scientific; MA, USA).

Samples were analyzed by LC-MS/MS on a Thermo Scientific Q Exactive+ Orbitrap Mass spectrometer coupled with a Proxeon Easy-nLC II HPLC (Thermo Scientific) Proxeonnanospray source. The peptides were separated using a 75 μm x 150 mm Magic C18 200Å 3U reverse phase column and a 120 min gradient with a flow rate of 300 nL/min. Data was acquired from 350-1600 *m/z* where the top ions were subjected to HCD (High Energy Collision Dissociation), and normalized collision energy of 27% was used for fragmentation. Tandem mass spectra were extracted by Proteome Discoverer version 1.2 charge state deconvolution and deisotoping were not performed. All MS/MS samples were analyzed using X! Tandem (The GMP group; https://www.thegpm.org/), which was set up to search the NCBI human refseq database and all-non-human common contaminants and an equal number of reverse sequences assuming the digestion of trypsin. Search allowed a fragment ion and a parent ion mass tolerance of 20 and 10 ppm, respectively. Scaffold v4.7.5 (Proteome Software Inc.; OR, USA) was used for data validation. Peptide identification was accepted if they achieved a threshold of 97% using Sacaffold’s LFDR algorithm, and protein identification was accepted if they contained at least two peptides at this threshold. Proteins sharing significant peptide evidence were grouped into clusters. Scaffold was also employed to identify the most abundant peptides of Transferon, which was based on the signal intensity (Total Ion Counts) and the probability of identification (>95%).

### Intact Mass Analysis

The intact mass of the Transferon peptides was determined by MS using the complete sequence of those proteins identified in the *de novo* sequencing. Eight Transferon batches (16E16, 16E15, 16F17, 16F18, 16J27, 16J28, 16J29, 16H15, and 16H26) were lyophilized and reconstituted in MS water (Honeywell; NJ, USA) at 2 mg/mL. Additionally, a sample of batch 18A01 was reduced and acetylated as previously described. A 10-μL volume of Transferon samples and recombinant human Ub (1μg/mL; Boston Biochem Inc.; MA, USA) was injected on a Vion^®^ ESI-IMS-Q-ToF coupled to an Acquity^®^ UPLC Class I system (Waters; MA, USA) and separated using a 1.7 µm CSH C18 (2.1 mm x 150 mm) column for identification and quantitation of Ub and a 3.0 µm C18 (2.1 x 150 mm) BioSuite^®^ column for verification of Ub identity (Both columns were purchased from Waters) at 60°C. Samples were eluted at 0.2 mL/min using water + formic acid (0.1%) (phase A) and acetonitrile + formic acid (0.1%) (phase B) as follows: 100% from 0 to 10 min of phase A, and a gradient of 100 to 50% from 10 to 85 min of phase A. Mobile phase reagents were MS grade and were purchased from Honeywell and Thermo Scientific. IMS-QTof was operated in positive polarity and sensitivity MS^E^/mode from 50 to 2000 *m/z*. Collision energies were 5.00 eV (low), 10.00 eV, (high), and 35.00 eV (high collision energy ramp end). Electrospray Ionization (ESI) parameters were set at 150°C source temperature, 450°C desolvation temperature, 0 L/h cone gas, 1,000 L/h desolvation gas, and 2.75 kV capillary voltage. A 50-pg/μL Leucine Enkephalin solution (Waters, 556.2766 *m/z*) was infused during the MS analysis at 5 μL/min for mass correction. Data were acquired and processed using Vion^®^ software in which complete sequence of those proteins identified in *de novo* sequencing was loaded, and all possible peptides derived from LysC/Trypsin in-specific digestion were searched with a 25 ppm tolerance; none chemical or posttranslational modifications were considered.

### Detection of Ubiquitin by ELISA

The presence of monomeric Ubiquitin (Ub) in Transferon was indirectly confirmed by indirect ELISA employing anti-Transferon antibodies and the kit OptEIA^®^ (BD Biosciences; CA, USA) according to the manufacturer’s instructions. Maxisorp ELISA plates (Nunc; Roskilde, Dinamarca) were coated with 50 μL of recombinant human Ub (10 μg/mL) in pH 9.5 carbonate buffer solution (BD Biosciences) at 4°C overnight. Ub detection was achieved using an in-house developed polyclonal anti-Transferon antibody (dilution 1:250) (Mellado-Sanchez et al., 2019) or a commercial polyclonal anti-Ub antibody (dilution 1:300; Boston Biochem Inc.), both developed in rabbit. Primary antibodies were detected using an anti-rabbit IgG antibody coupled to Horseradish Peroxidase (dilution 1:2,500; Sigma-Aldrich). Data were analyzed at 450 nm/570 nm in an Epoch^®^ spectrophotometer (BioTek^®^; VT, USA) after performing the colorimetric reaction with a 3,3’,5,5’ Tetramethylbenzidine (TMB) (BD Biosciencs). Data were processed using the Gen5 software (BioTek^®^).

### Identification of Ub Using Anti-Ub Polyclonal Antibodies and MS

The total Ub content of Transferon was removed using an anti-Ub polyclonal antibody (Boston Biochem Inc.) and used as a negative control in the *in vivo* assay. Transferon sample (batch 19J20) were lyophilized and reconstituted in water for injection (Pisa Laboratories) at 2 mg/mL. A 200 μL volume was incubated with 2 μL of anti-Ub polyclonal antibody (Boston Biochem Inc.) at 4°C overnight. Then, the sample was filtrated by centrifugation through a 50 kDa membrane (Millipore; MA, USA); the permeate and the retained fractions were analyzed by the MS intact method to confirm the removal of Ub. The permeate was sterilized by filtration using a 0.22 μm membrane (Millipore) and stored at 4°C until use.

### Comparison of Ub and Ub(-GG) Structures by Homology Modeling

Ub and Ub(-GG) were modeled to determine the effect of the lack of two-terminal Gly in their structure. A protein structure homology modeling was applied using SWISS-MODEL (Guex et al., 2009; Bertoni et al., 2017) based on the 2jwz.1.A structure, which was obtained by nuclear magnetic resonance (NMR) and shared a 94.74% of homology with Ub.

### Quantitation of Total Ub by MS

The total concentration of Ub [Ub+Ub(-GG)] was calculated in eight Transferon batches (18A01, 18A02, 18A03, 18A04, 18B05, 18E13, 18E14, and 18E16) by MS. Transferon samples were lyophilized, reconstituted with 100 µL of MS water (Honeywell) and added with 10 μL of 32 pmol/μL Glu-1-fibrinopeptide B (Glufib) as an internal standard. Samples were analyzed using the intact mass MS method, as previously described. The deconvoluted area of Ub and Ub(-GG) was obtained, added and corrected vs. Glufib response. The total Ub concentration was calculated using a recombinant human Ub (Boston Biochem Inc.) standard curve also prepared with Glufib. Data were acquired and processed with the UNIFI^®^ software.

### HSV-1 Infection Murine Model

The relevance of the Ub content in Transferon was evaluated using an HSV-1 infection murine model, as previously described by Salinas-Jazmin and cols. (Salinas-Jazmin et al., 2015); trained researchers blindly performed all procedures. Briefly, 4-week-old and 14–18 g weigh male BALB/c mice (Ferandelh; Mexico City, Mexico) were shaved on the back and anaesthetized with 10 μL/g of 6.4 mg/mL sodium pentobarbital (Pisa Laboratories) *via* intraperitoneal. Mice had *ad libitum* access to standard chow (Harlan Labs; IN, USA) and filtered water. They were housed in a P/NC IVC system (Allentown Inc.; NJ, USA) at 22°C/55% relative humidity and 12-h light/dark cycle. After 24 h of infection, Day 0, mice were inoculated by cutaneous scarification with 10 μL of 5 × 10^6^ PFU/mL of Herpes simplex virus type 1 (HSV-1) Koss strain, previously expanded in African Green Monkey Kidney cells (Vero^®^ CCL-81) and Eagle’s minimal essential medium (EMEM) supplemented with 10% of fetal bovine serum, all acquired from ATCC (VA, USA). On day 2, infected mice were grouped (n = 9) and ORO administered with the next treatments: Transferon (0.75 μg/200 μL), Transferon (0.75 μg/200 μL) + Ub (0.75 μg/200 μL), Ub-depleted Transferon (≈0.75 μg/200 μL), and Ub (0.75 μg/200 μL). Recombinant human Ub (Boston Biochem Inc.) was employed. Groups received the same treatment every other day until day 10 and were maintained under observation until day 20 along with a non-infected/non-treated control (negative control) and infected/non-treated (infection control). During the assay, mice were daily monitored to identify infection-associated symptoms, such as paralysis of the lower extremities, reduced mobility, and weight loss. Animals were euthanized if they experienced a total loss of mobility; these events were counted as deaths. All procedures were performed according to Mexican and International Guides for Care and Use of Laboratory Animals (Secretaría de Agricultura, 1999; Animals, 2011) and approved by the Ethical Committee of the Transfer Factor Project (protocol FTU/IB/012/010/PRO) (Salinas-Jazmin et al., 2015). All efforts were made to minimize animal suffering.

### Statistics

The percentage contribution of proteins to the total mass spectrum of Transferon and their percentage of coverage are reported as the average value (n = 10). In both cases, the standard deviation (SD) bar is provided. The hit number per peptide is also reported as the average of the 10-batch analysis. In the ELISA assay, a D’Agostino-Pearson omnibus normality test was performed to determine normal distribution in the study groups, then a Kruskal-Wallis test followed by a Dunn’s multiple-comparison test was applied to determine statistical difference among groups. The percentage of survival is presented using a Kaplan-Meier survival plot. All these statistical analyzes were performed using GraphPad Prism, version 6.00 for Windows (GraphPad Software, La Jolla CA, USA).

## Results

### MS Sequencing Analysis

**Figure 1:**
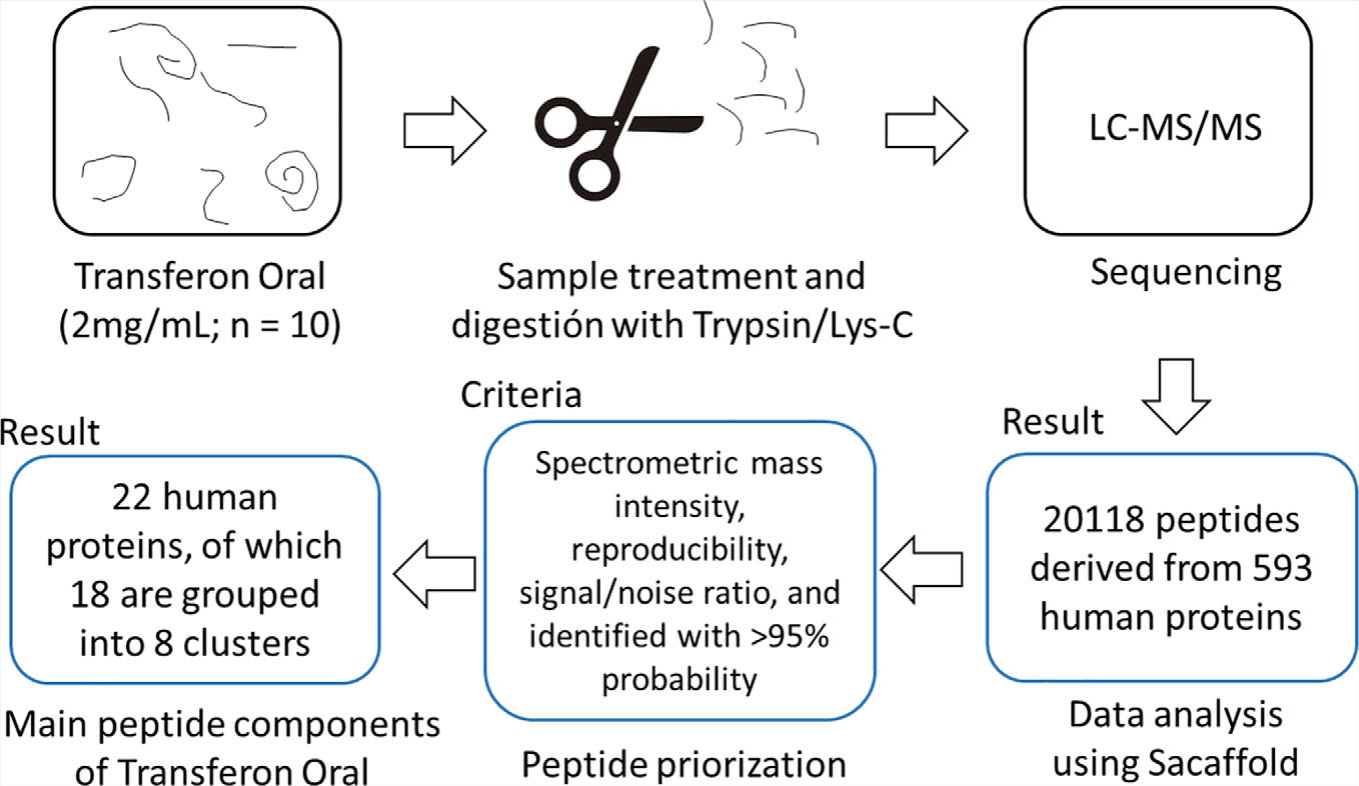
Sequencing analysis of Transferon by MS. Transferon peptides were digested using Trypsin/Lys-C and sequenced by LC-MS/MS. The obtained peptide mass spectrum were prioritized based on its mass intensity, reproducibility among batches, signal and identification reliability. The batch-to-batch reproducible peptides of Transferon come from 22 proteins.

**Figure 2:**
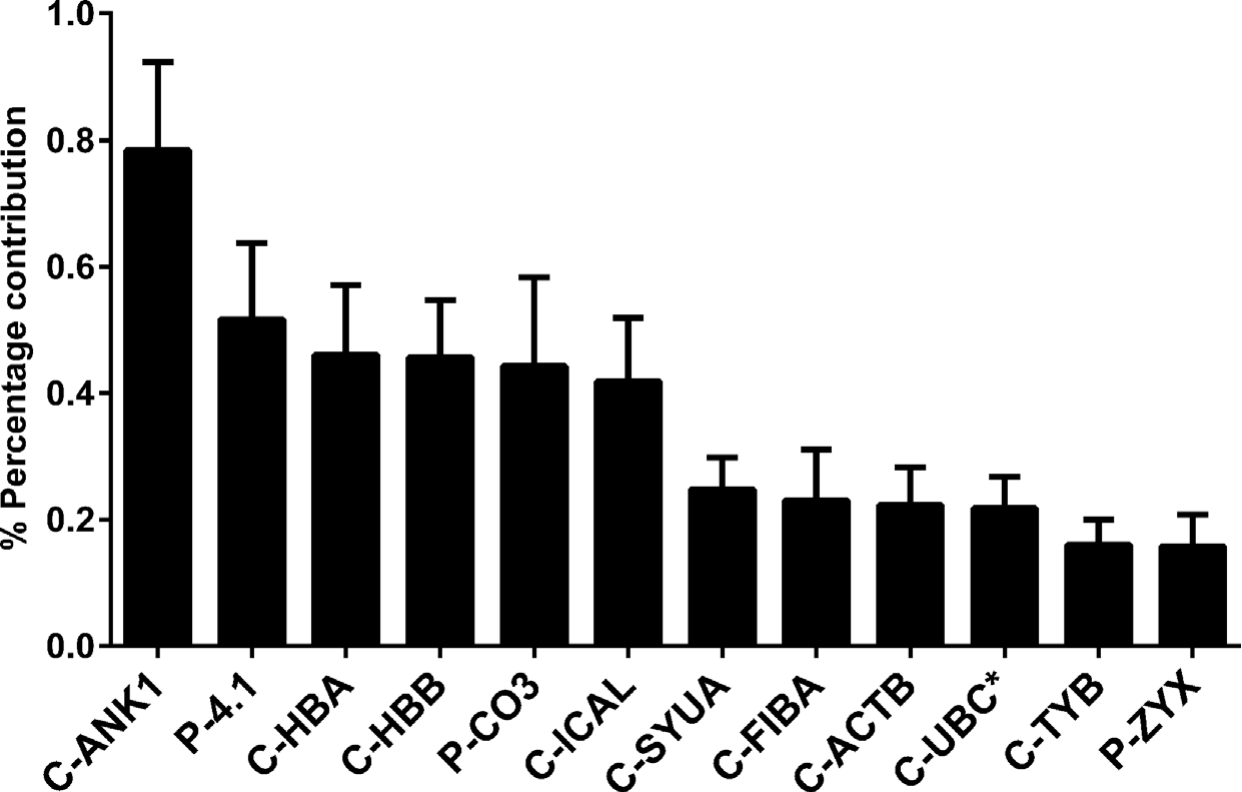
Percentage of the contribution of proteins to the mass spectrum of Transferon. The relevance of proteins that consistently provide peptides to Transferon was determined based on their the average total mass spectrum obtained in the 10-batch sequencing analysis. The cluster ANK-1 contributes the most to the mass spectra of Transferon. The bars indicate standard deviation (SD) of the analysis. *, monomer. Clusters: ankyrin (ANK-1), hemoglobin subunit α (HBA), hemoglobin subunit β (HBB), calpastatin (ICAL), α-synuclein (SYUA), fibrinogen α-chain (FIBA), cytoplasmic actin 1 (ACTB), polyubiquitin C (UBC), thymosin (TYB); Proteins: protein 4.1 (4.1), complement C3 protein (CO3), and zyxin (ZYX).

A two-stage mass spectrometric analysis was performed to determine the most abundant peptide component of Transferon. In the first stage, 10 batches of Transferon were *de novo* sequenced using a trypsinization method (**Figure 1**). In this analysis, it was found that each Transferon sample is made up of around 20,000 peptides derived from 593 human proteins. These results were analyzed to describe the peptide composition of Transferon qualitatively. In this sense, the protein clusters were ordered from highest to lowest spectrometric intensity, an selected those that i) appeared consistently among the 10 batches analyzed (reproducibility), ii) were identified with a probability higher than 95%, and iii) whose spectrometric intensity was above the signal/noise ratio, which was defined as the point where sequences from keratin, a common environmental pollutant in this type of analysis, were detected (Hodge et al., 2013). It was determined that the most abundant Transferon peptides, based on the intensity of their mass spectrum, come from 22 human proteins, 18 of them grouped in clusters (**Table 1**). Overall, these peptides account for the 4,319% (SD < 1.03) of the total peptide content of Transferon, being the ankyrin (ANK-1) peptides that contribute the most (**Figure 2**).

**Table 1:** Main proteins than provide of peptide components to Transferon.

	**Protein or cluster**	**Protein accession number**	**Percentage of total Transferon oral spectrum (%)**	**SD**	**Link to access the full sequence**
**C1**	Cluster of Ankyrin-1	1) ANK1_HUMAN 2) Q6PK32_HUMAN (ANK1_Fragment)	0.784	0.14	https://www.uniprot.org/uniprot/P16157 https://www.uniprot.org/uniprot/Q6PK32
**P2**	Protein 4.1	3) 41_HUMAN	0.517	0.12	https://www.uniprot.org/uniprot/P11171
**C3**	Cluster of Haemoglobin subunit α	4) HBA_HUMAN 5) G3V1N2_HUMAN (HBA_Fragment)	0.461	0.11	https://www.uniprot.org/uniprot/P69905 https://www.uniprot.org/uniprot/G3V1N2
**C4**	Cluster of Haemoglobin subunit β	6) HBB_HUMAN 7) E9PEW8_HUMAN (HBB_Fragment)	0.457	0.09	https://www.uniprot.org/uniprot/P68871 https://www.uniprot.org/uniprot/E9PEW8
**P5**	Complement 3 protein (C3)	8) CO3_HUMAN	0.443	0.14	https://www.uniprot.org/uniprot/P01024
**C6**	Cluster of Calpastatin	9) ICAL_HUMAN 10) A0A0A0MR45_HUMAN (ICAL_Fragment 11) D6RC54_HUMAN (ICAL_Fragment 2)	0.419	0.10	https://www.uniprot.org/uniprot/P20810 https://www.uniprot.org/uniprot/A0A0A0MR45 https://www.uniprot.org/uniprot/D6RC54
**C7**	Cluster of α-synuclein	12) SYUA_HUMAN 13) H6UYS7_HUMAN (SYUA_Fragment)	0.248	0.05	https://www.uniprot.org/uniprot/P37840 https://www.uniprot.org/uniprot/H6UYS7
**P8**	Fibrinogen α-chain	14) FIBA_HUMAN	0.231	0.08	https://www.uniprot.org/uniprot/P02671
**C9**	Cluster of Actin, cytoplasmic 1	15) ACTB_HUMAN 16) I3L3R2_HUMAN (ACTB_Fragment) 17) POTEE_HUMAN	0.223	0.06	https://www.uniprot.org/uniprot/P60709 https://www.uniprot.org/uniprot/I3L3R2 https://www.uniprot.org/uniprot/Q6S8J3
**C10**	Cluster of Polyubiquitin-C (monomer)	18) UBC_HUMAN 19) J3QRK5_HUMAN (UBC_Fragment)	0.218	0.05	https://www.uniprot.org/uniprot/P0CG48 https://www.uniprot.org/uniprot/J3QRK5
**C11**	Cluster of Thymosin	20) TYB4_HUMAN 21) TYB10_HUMAN	0.160	0.04	https://www.uniprot.org/uniprot/P62328 https://www.uniprot.org/uniprot/P63313
**P12**	Zyxin	22) ZYX_HUMAN	0.158	0.05	https://www.uniprot.org/uniprot/Q15942
**Global values**			**4.319**	**1.03**	

These proteins were identified in the MS sequencing analysis using Trypsin/LysC digestion and are presented in decreasing order according to their total spectrometric intensity. The SD obtained in the 10-batch sequencing analysis is also provided. C, cluster; P, protein.

**Figure 3:**
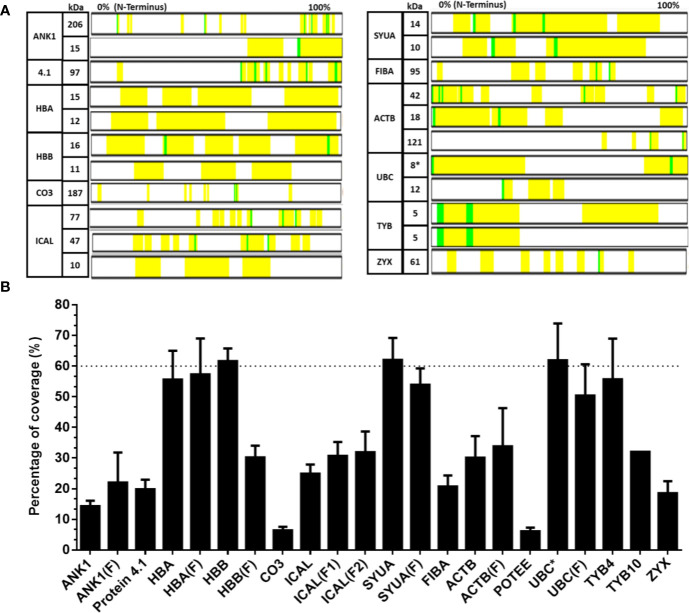
Analysis of the percent coverage of the proteins that consistently provide peptides to Transferon. The proteins that consistently provide peptides to Transferon were individually analyzed, and their total contribution was reported as the percent of coverage. **(A)** The map of the peptides found in the sequencing analysis of batch 14G18 is showed as an example; the identified peptides are marked in yellow and in green chemical modification. Note that the length of the proteins was normalized and does not represent its real size. **(B)** Average percentage coverage per protein obtained in the 10-batch sequencing analysis. The average percent of coverage of HBB, SYUA and monomer UBC was 61.6, 62.0, and 62.2%, respectively. The bars indicate the SD of the 10-batch analysis. C-, cluster; (F), fragment; *, monomer; ankyrin (ANK-1), hemoglobin subunit α (HBA), hemoglobin subunit β (HBB), complement C3 protein (CO3), calpastatin (ICAL), α-synuclein (SYUA), fibrinogen α-chain (FIBA), cytoplasmic actin 1 (ACTB), POTE ankyrin domain family member E (POTEE), polyubiquitin C (UBC), thymosin β4 (TYB4), thymosin β10 (TYB10), and zyxin (ZYX).

**Figure 4:**
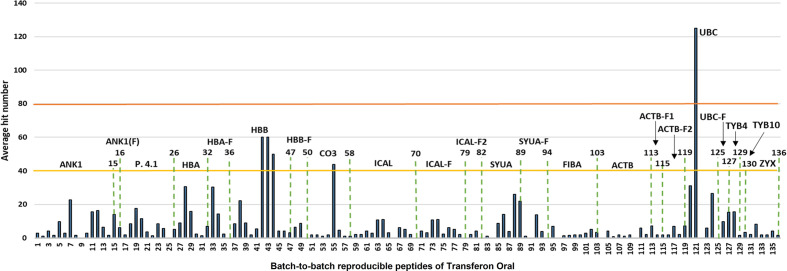
Analysis of the abundance of batch-to-batch reproducible Transferon peptides. The reproducible peptides of Transferon were defined as those identified in at least 8 of 10 sequenced batches. Then, their average hit number was obtained and used as an abundance indicator. It was determined that ankyrin (ANK-1) provide to Transferon the higher number of consistent peptides (15 peptides). On the other hand, polyubiquitin-C (UBC) provides 6 peptides (from 120 to 125), but one of them (121) with the higher average hit number (125).

The contribution of each protein to the mass spectrum of Transferon depends on: i) the number of peptides provided to the mixture and ii) the spectral intensity of each peptide. Thus, the relative abundance per protein in Transferon was qualitatively calculated based on the percentage of coverage and the average hit number of their peptides. The coverage map of Transferon evinced that its batch-to-batch reproducible peptides come from different protein regions: C-Terminal end (ankyrin-1), N-Terminal end (actin, cytoplasmic 1), and, in some cases, from the whole protein (hemoglobin subunit β and thymosin clusters) (**Figure 3A**). The percentage of coverage analysis revealed that ANK-1, a 206-kDa protein, contributes with 14% of its sequence to Transferon, whereas low–molecular mass protein clusters provide with more than 60%, i.e., hemoglobin subunit β (16 kDa), α-synuclein (14 kDa), and polyubiquitin-C (17 kDa) (**Figure 3B**). Further, it was obtained the average number of hits per peptide as an indicator of relative abundance. At this regard, the peptides that appeared in at least eight out of 10 sequenced batches were selected. One hundred thirty-six relevant peptides were identified, and their average number of hits were grouped in a high (>80 hits) or intermediate (40–80) hit region (**Figure 4**). It was found that the high contribution of ANK-1 to the mass spectrum of Transferon is because this cluster contributes the highest number of peptides, 17 in total. In counterpart, a peptide from the polyubiquitin-C (UBC) cluster had the maximum number of hits (125) although this cluster has a lower contribution to the Transferon spectrum.

### Identification of Ub and Ub(-GG) in Transferon

**Figure 5:**
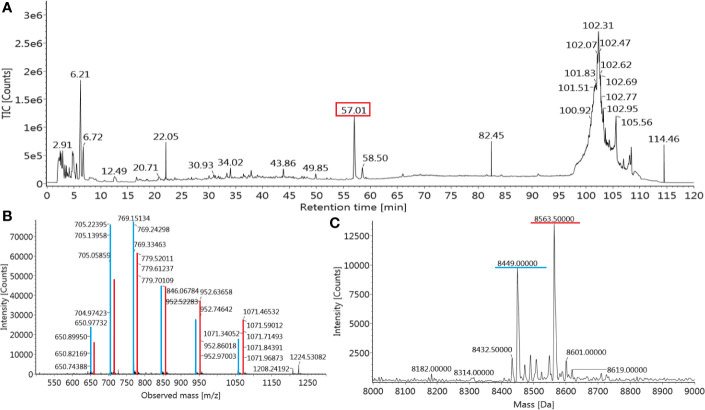
Identification of monomeric Ub in Transferon. **(A)** Transferon (Batch 18A01; 2 mg/mL) was analyzed by ESI-IMS-Q-ToF using an intact mass approach with a small-particle size column (CSH C18, 1.7 μm). The most intense *m/z* signal of the total ion counting (TIC) profile was observed at 57.01 min (red square). **(B)** The *m/z* profile and **(C)** the deconvoluted mass of the main Transferon TIC signal (marked in red) was similar to the theoretical values of monomeric Ub. A second peptide entity with a mass close to monomeric Ub (8449.0 Da) was also observed in the main chromatographic peak (*m/z* transitions in blue).

**Figure 6:**
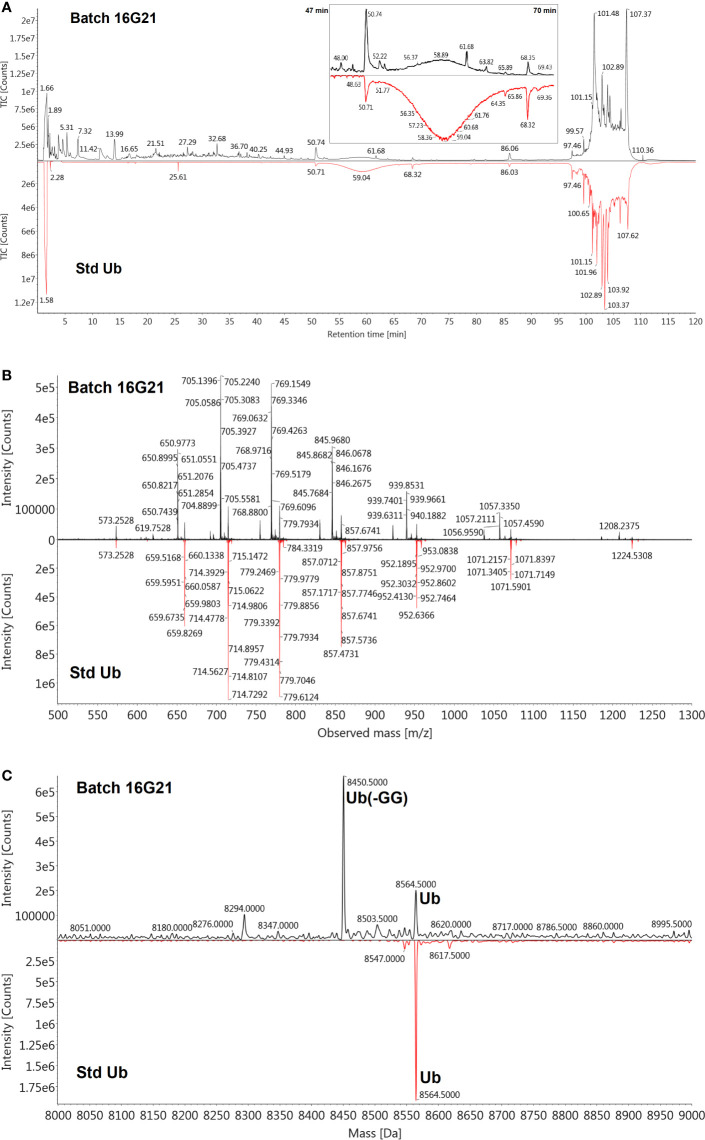
Verification of the presence of Ub in Transferon and identification of Ub (-GG). **(A)** A monomeric standard of Ub (Std Ub) was analyzed by MS using a 3.0 µm BioSuite^®^ C18 column, along with the Transferon batch 16G21; both TIC profiles were similar from 47 to 70 min. **(B)** This segment of the TIC profile was combined and the *m/z* signals between Transferon and SD Ub were also similar when compared in a mirror projection. **(C)** The deconvolution of the *m/z* profiles of Transferon exhibited two masses, the firs (8564.5 Da) corresponded to that of the Std Ub (8564.5 Da), whereas the second (8450.5 Da) to the theoretical mass value of monomeric Ub and monomeric Ub without two-terminal Gly [Ub(-GG); 8,450.74 Da].

The *de novo* sequencing of Transferon allowed to i) identify the proteins that mainly contribute to its peptide fraction, ii) and to qualitatively describe its peptide content. However, it is not useful to identify Transferon peptides with a relevant biological activity because their identification was biased by the trypsinization procedure used during sample processing. In the second stage of MS characterization, an analysis of the intact mass of Transferon was performed using the whole sequence of the cluster identified in phase 1. In this analysis, the highest total ion counting (TIC) signal was observed around 57 min (**Figure 5A**) using a 1.7-μm CSH C18 column. Using the MaxEnt1 algorithm the 57 min signal was combined, and the obtained *m/z* and its deconvoluted mass (**Figures 5B, C**, respectively) were very similar to the theoretical mass of monomeric Ub (8564.84 Da). Interestingly, in the same chromatographic peak, a second peptide signal with a similar mass to monomeric Ub was observed (8449.0 kDa). The presence of monomeric Ub in Transferon was verified by analyzing a recombinant human monomeric Ub standard and Transferon side-by-side using a 3.0-µ C18 BioSuite^®^ column; both samples exhibited the same TIC profile at the elution time of monomeric Ub, i.e., from 50 to 69 min (**Figure 6A**). The combination of the TIC profile of Transferon from 50 to 69 min evinced two sets of signals, as previously observed using the small-size particle CSH C18 column, one of them was similar to the standard of monomeric Ub (**Figure 6B**). After deconvolution by a MaxEnt1 algorithm, these sets of *m/z* signals of Transferon were assigned to the monomeric Ub (8564.50 Da) according to the intact mass of the Ub standard, and the second set, the most intense, to an Ub-related structure that lacks the two terminal glycine [Ub(-GG); 8450.5 kDa] according to the predicted sequence by the UNIPROT software and to the theoretical mass calculation of Ub(-GG) using the ExPASy tool (8450.74 Da) (**Figure 6C**). A reduced sample of Transferon was analyzed under the same analytical conditions to confirm that the *m/z* signals attributable to Ub and Ub (-GG) instead of peptide aggregates. The reduced sample also showed the Ub and Ub (-GG) *m/z* patterns (**Figure S1**). Further, the TIC and *m/z* profiles of Ub and Ub(-GG) were reproducible in 8-batch Transferon MS analysis (**Figure S2**).

**Figure 7:**
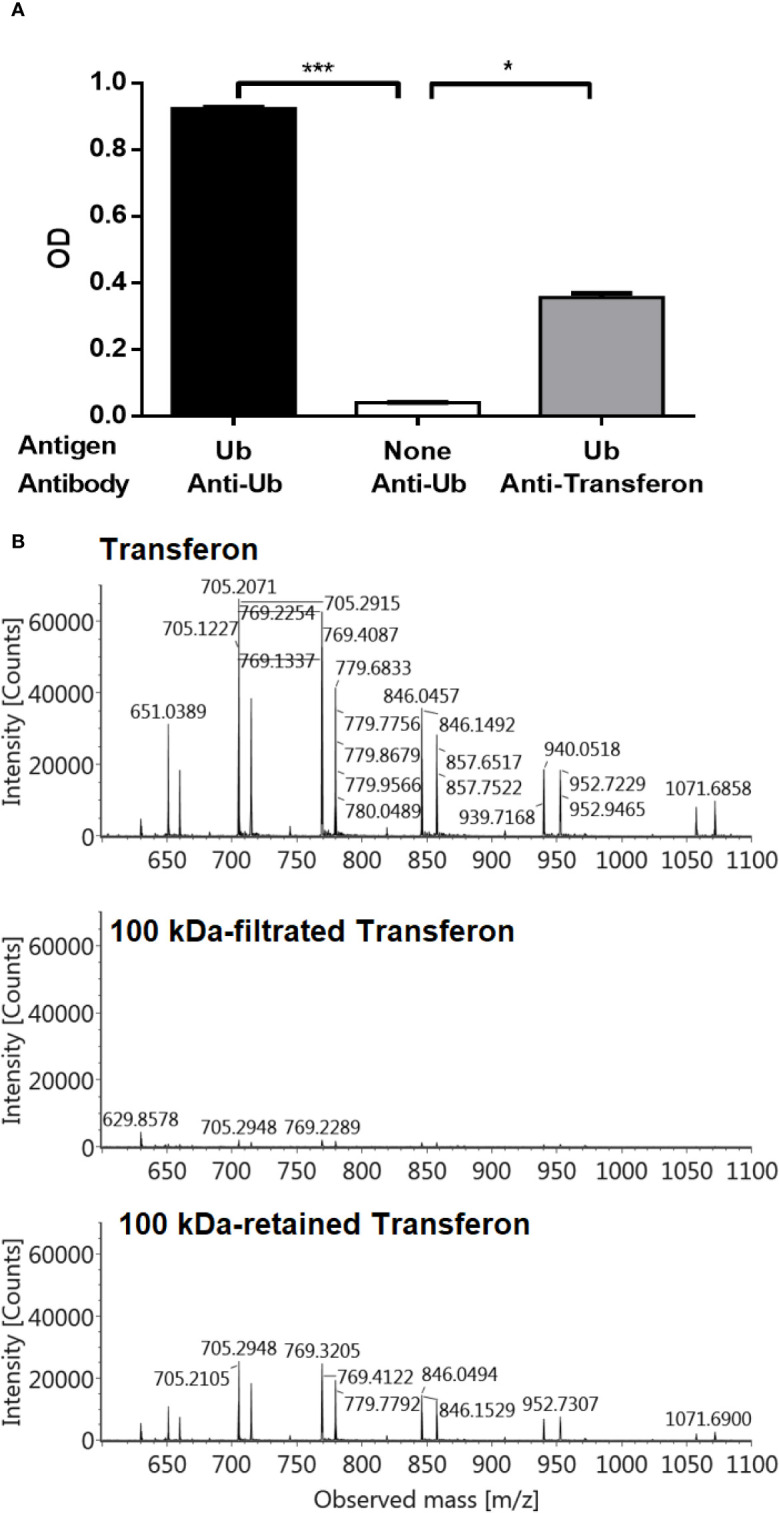
Analysis of Ub and Ub(-GG) using polyclonal antibodies. **(A)** The presence of Ub in Transferon was indirectly confirmed using an anti-Transferon polyclonal antibody developed in rabbit by in-house methods (Mellado-Sanchez et al., 2019), which turned out to recognize monomeric Ub. The SD is presented per group. **(B)** Also, a 2 mg/mL of Transferon sample (Batch 19J20) was overnight incubated with a commercial anti-Ub polyclonal antibody (1:100 dilution) and filtrated through 100 kDa membrane. Filtrated and retained fractions were analyzed by MS. SD bars are indicated per group. OD, optical density; TMB, Tetramethylbenzidine; C, control. H = 15.6; (d*f* = 2, N = 18); ***P < 0.001; *P < 0.05.

**Figure 8:**
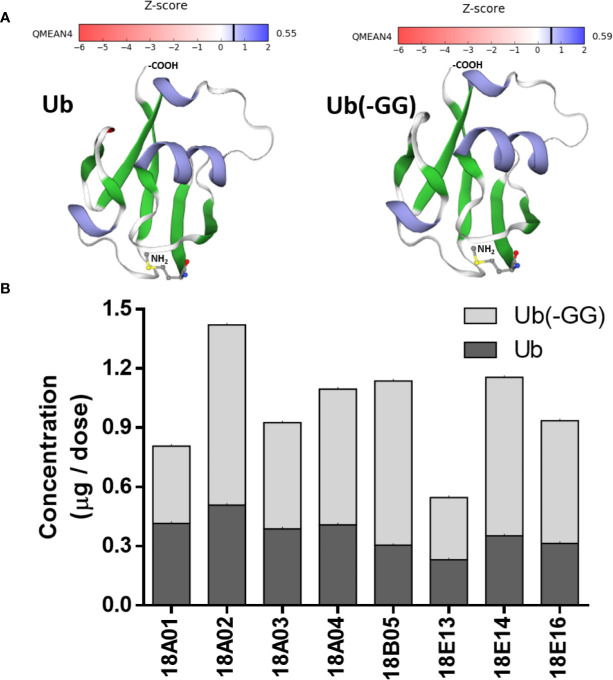
Structural comparison of Ub and Ub(-GG) and total Ub quantitation in Transferon. **(A)** It was evaluated the impact of the lack of the terminal Gly on the structure of Ub using the SWISS-MODEL homology-modeling tool. Ub and Ub(-GG) were considered the same as they have similar shapes and Z-scores. **(B)** In this sense, the total Ub content of eight Transferon batches was quantitated per dose (5 mL) using the intact mass method. The average concentrations were 0.366 μg/dose (CV, 22.97%), 0.637 μg/dose (CV, 33.46%), and 1.003 μg/dose for monomeric Ub, Ub(-GG) and total Ub, respectively.

The identity of Ub and Ub(-GG) in Transferon was also determined using polyclonal antibodies. A polyclonal anti-Transferon antibody, which was generated by our group (Mellado-Sanchez et al., 2019), recognized recombinant human monomeric Ub, as well as a commercial polyclonal anti-Ub antibody (**Figure 7A**). Besides, a sample of Transferon (2 mg/mL) was incubated with a commercial polyclonal anti-Ub antibody at 4°C overnight and filtered through a 100 kDa membrane. The analysis of intact mass showed that the anti-Ub antibody retained both Ub and Ub(-GG) during filtration (**Figure 7B**). This result also confirmed that the two sets of *m/z* signals that co-elute at 55 min in the TIC profile of Transferon correspond to Ub and Ub (-GG), two structurally and physicochemically similar peptide entities. The structural similarity between Ub and Ub(-GG) was also evaluated by homology-modeling using the SWISS-MODEL tool. The models obtained for Ub and Ub(-GG) and their Z-score values were similar (0.55 vs. 0.59) (**Figure 8A**). Taking all these results into account, it was considered the Ub and the Ub(-GG) as the same entity (total Ub). In this sense, the total Ub was quantified in eight Transferon batches. A 5-mL vial (0.4 mg/mL of Transferon, equivalent to one dose) was lyophilized, reconstituted in water (2 mg/mL), and quantified by MS using a standard human monomeric Ub curve; the results were calculated in μg/dose, which is equivalent to μg/5 mL. Quantification showed that the average content of the Ub(-GG) peptide almost doubles that of monomeric Ub (0.637 μg/dose vs. 0.366 μg/dose) in Transferon and that the average content of total Ub is 1,003 μg/dose (**Figure 8B**).

### Evaluation of the Functional Relevance of Ub as the Main Peptide Component of Transferon

**Figure 9:**
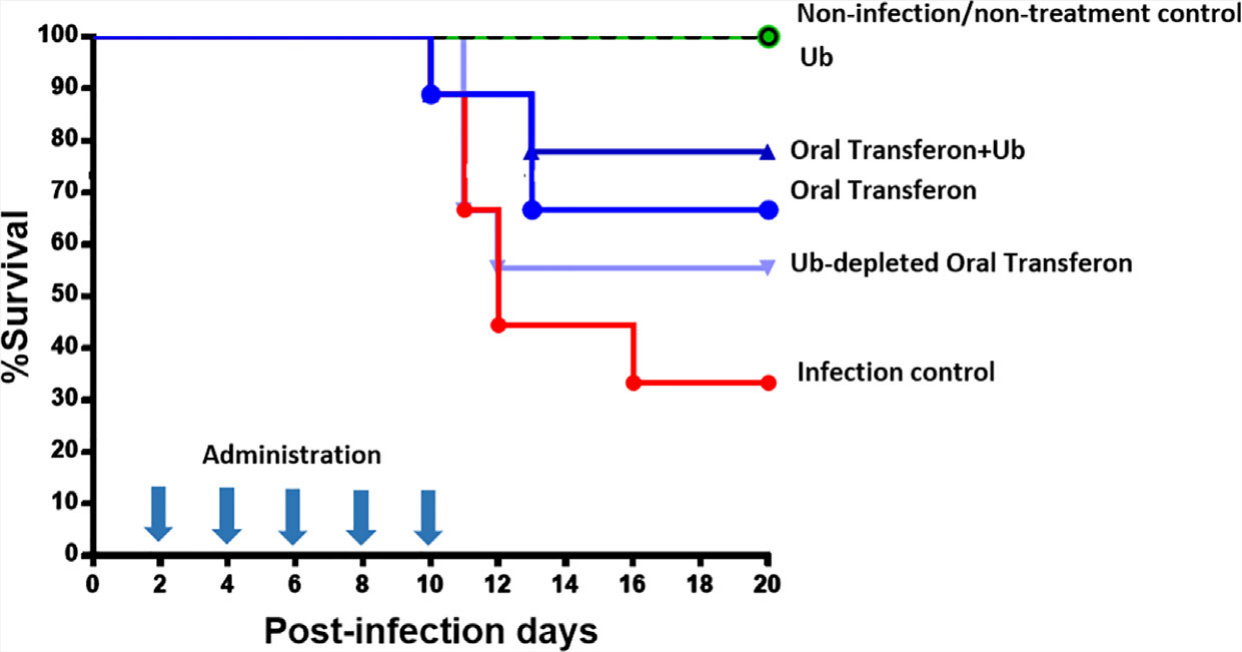
Effect of Transferon and Ub in the HSV-1 infection murine model. HSV-1 infected mice were treated with Transferon (0.50 μg/200 μL), monomeric Ub (0.75 μg/200 μL), “Transferon Oral” + monomeric Ub, or monomeric Ub-depleted Transferon (≈0.50 μg/200 μL). The addition of Ub to Transferon increased (77.7 vs. 66.6%), and the removal of Ub decreased (55.5 vs. 66.6%) the %survival respect to the group treated only with Transferon. Ub treated group showed 100% of survival.

A murine model of HSV-1 infection was used to assess the biological relevance of Ub in Transferon. In this model, BALB/c mice are infected with HSV-1, pharmacological treatment is ORO administered at 2, 4, 6, 8, and 10 days post-infection, and remain under observation for an additional 10 days to assess survival percentage. The administered treatments were Transferon (0.50 μg/200 μL); Transferon added with monomeric Ub (0.50 μg/200 μL + 0.75 μg/200 μL, respectively); Ub-depleted Transferon (≈0.50 µg/200 µL), which was removed with a commercial polyclonal anti-Ub antibody (**Figure 7B**); and Ub (0.75 μg/200 μL) (**Figure 9**). In this assay, it was observed that the group treated with Transferon + Ub showed an increase in the percentage of survival compared to the group treated with Transferon only (77.7 vs. 66.6%), whereas the percentage of survival decreased in the group treated with Ub-depleted Transferon compared to Transferon group (55.5 vs. 66.6%). Interestingly, the group treated with Ub alone showed 100% survival, as did the negative control group (no infection/no treatment). The concentration of Ub used for this assay was determined in a previous assay where it was observed that concentrations equivalent to less than 50% of Transferon did not induce significant changes in survival compared to the infection control (data not shown). Altogether, these results indicate that monomeric Ub is a major active component of the peptide mixture of Transferon.

## Discussion

Transferon is an extract obtained by the dialysis/filtration of human buffy coats, and its API is a complex mixture of peptides of less than 10 kDa in size with a reproducible composition between batches (Medina-Rivero et al., 2016). This blood-derived product, which is marketed as an oral product (Transferon Oral) or as an injectable formulation (Transferon^®^) when added with glycine and sucrose, is used in the treatment of diseases with an inflammatory component (Estrada-Parra et al., 1998; Medina-Rivero et al., 2014). Preclinical and clinical information indicates that Transferon modulates the production of pro-inflammatory cytokines and improves the resolution of infectious diseases. However, the molecular bases of the therapeutic effects have not been fully understood, since it is not clear how the same product can induce Th1 or Th2 mechanisms depending on the condition in which it is used (Jimenez-Uribe et al., 2019). The immune “buffering” effect of dialyzable leukocyte extracts relay on their composition: there are regulatory molecules that control hypersensitivity and allergies, and an inducing fraction that reinforce the antigenic stimulus (Tulina et al., 2019).

Transferon is a complex biological drug composed of multiple peptide components. It is important to note that the mechanism of action of a complex drug is almost impossible to determine because it is the sum of the biological effects and signaling pathways activated by the multiple components of the drug during its residence time in the body. Notwithstanding, identifying the most abundant peptide component of Transferon, and the signaling pathways associated with it, will provide valuable information for better rational use and to develop new drugs with improved characteristics.

In this work, monomeric Ub was identified as the most abundant peptide component of the peptide mixture of Transferon based on its relative ionization intensity in a set of UPLC-MS analysis. In addition, we evinced that monomeric Ub emulated the protective effect of the whole peptide mixture in an HSV-1 murine infection model when ORO administered. Taking into account that Transferon is a complex biological drug, all analyses were performed using 10 batches for reproducibility purposes.

### Sequencing Analysis of the Peptide Components of Transferon

To the best of the authors’ knowledge, there is only one report on the identification of peptide sequences in dialyzable extracts derived from mammalian cells (mice and bovine) in 2000 (Kirkpatrick, 2000). In this work, Kirkpatrick obtained the sequence of conserved peptides by Edman’s degradation sequencing. However, those peptide sequences have not been found in any known proteome to date. Although Edman degradation is still the only actual sequencing technique today, its effectiveness is limited to high purity peptide samples (Fricker, 2015). Thus, this technique is not suitable for the analysis of DLE because these products are composed of a myriad of peptides with a low relative concentration. Nowadays, identification and quantitation of a vast number of peptides in complex samples are performed by different high-throughput methods, all based on MS (Schrader, 2018; Azkargorta et al., 2020; Pourjoula et al., 2020). In this sense, we proposed the use of high-resolution MS techniques coupled to high-performance liquid chromatography (UPLC) for the identification of the most abundant peptide component of Transferon. Considering that MS deduces the sequence of peptides based on spectral interpretation, we took into account the peptides identified with a high probability (>95%) and narrowed the analysis to human peptides.

The identification of the peptide components of Transferon was performed in two stages: a *de novo* sequencing and an intact mass sequencing. In the first stage, the 12 protein clusters from which the reproducible peptides between batches (4,319% of the total mass spectrum) were identified, being the ANK-1 cluster the most relevant. These protein clusters come from human leukocytes and erythrocytes. They can be grouped into: i) structural membrane proteins (Ankyrin, protein 4.1, Zyxin and α-synuclein) and intracellular structural proteins (Actin-1 and thymosin β4), ii) functional intracellular proteins (hemoglobin α and β subunit, polyubiquitin C, and calpastatin) and plasma soluble proteins (fibrinogen α chain and complement C3 protein). Among them, some proteins directly affect the immune system: C3 protein plays a central role in the complement system cascade, a mechanism of the innate immune system (Vignesh et al., 2017); peptides derived from the α-chain of fibrinogen elicit a lymphocyte suppressive functions (Plow and Edgington, 1986); extracellular hemoglobin (α and β chains) act as pathogen-associated molecular patterns (PAMP) and activates Toll-like receptors and, when oxidized, functions as a damage-associated molecular patterns (DAMP) (Lee and Ding, 2013); extracellular Ubiquitin has pleiotropic effects in inducing cytokine secretion and counteracts the inflammatory effect of DAMP (Patel et al., 2006; Majetschak, 2011), whereas thymosin β4 regulates the inflammatory response derived from damage (Qiu et al., 2011). The nature of protein clusters that provide the most abundant peptides suggests that the main mechanisms of Transferon are related to the induction and regulation of DAMP signaling pathways and the innate immune system. However, extensive research is required to determine whether the peptides identified in Transferon retain the biological functions of the complete proteins from which they are derived.

Sequence coverage analysis showed that protein clusters provide peptides from different regions of their structure, in some from their C-terminal, N-terminal or along the entire protein sequence. This suggests that in the cell lysis stage by freeze/thaw cycles, during the manufacturing process of Transferon, proteases from leukocytes are released into the medium and digest the exposed cellular components in a random but controlled pattern. This process resembles the stage of polymerization and acid digestion of glatiramer acetate peptides, where up to 10^36^ peptides of an unknown sequence are generated, but with a consistent peptide polydispersion among batches (Campos-Garcia et al., 2017).

After analyzing the clusters and the proteins from which Transferon is generated, the peptides were individually evaluated. In this analysis, 136 reproducible peptides were identified among batches, and their relevance (abundance) was determined based on their average number of hits obtained in *de novo* sequencing of Transferon batches. It was determined that the spectral abundance of ANK-1 is because this cluster contributes with the highest variety of peptides (17 different peptides) to Transferon compared to the rest of the clusters. In contrast, the clusters of polyubiquitn-C, hemoglobin β subunit and protein C3 provide fewer peptide sequences but with a higher frequency (number of hits) per batch. Additionally, the high coverage percentage of low–molecular mass proteins (<15 kDa), such as polyubiquitin C (monomer), hemoglobin β subunit, and α-synuclein suggested that they could be complete in Transferon.

The disadvantage of *de novo* sequencing is that the sequence obtained from the peptides is biased by trypsin digestion during sample preparation. For this reason, the second stage of spectrometric analysis consisted of identifying the intact peptides. In this analysis, it was observed that the most intense signal of Transferon is due to two interrelated low–molecular mass proteins: the Ub monomer (8.56 kDa) and a Ub variant that lacks its two terminal Gly [Ub(-GG); 8.45 kDa]. This observation correlated with the analysis of the number of hits where it was determined that the most frequent peptide come from the polyubiquitin-C cluster, and with the coverage map that suggested that the Ub monomer could be complete in Transferon. Remarkably, the analysis of intact mass showed that the monomeric Ub and its variant Ub(-GG) are the only complete proteins in Transferon, which indicates these structures resist enzymatic hydrolysis from its release, mainly from erythrocytes, to the finished product (Patel et al., 2006). This finding is based on the fact that the monomeric Ub is a natural serum globular protein of small size (76-amino-acid) that lacks posttranslational modifications and possesses high thermal, structural, and proteolytic stability (Job et al., 2015).

It is important to note that the identity of Ub was verified using and Ub standard by UPLC-MS in a straightforward way. On the other hand, the Ub(-GG) identity is assigned to the 8450.5-Da mass because of its high similarity to its theoretical mass (8450.74 Da), and indirectly by comparing its physicochemical behavior to that elicited by Ub: i) both m/z signals are recognized by commercial anti-Ub antibodies, which indicates similar sequences; ii) their m/z patterns are similar, which evinces a similar content of ionizable groups; and iii) both structures co-elute in the UPLC-MS analysis, which indicates that have similar hydrophobic properties. This evidence highly suggests that the mass 8450.5 Da corresponds to Ub(-GG). Notwithstanding, amino acid substitution in the primary sequence cannot be discarded in this work. In this sense, we will corroborate the full sequence of Ub(-GG) in further research.

### Ub as a Probe to Infer One Mechanism of Action of Transferon

We evaluated the biological relevance of Ub and Ub(-GG) for Transferon using an HSV-1 infection murine model. For this purpose, we considered Ub and Ub(-GG) the same molecule (total Ub) considering that are physicochemical and structurally similar and that the lack of terminal Gly does not affect the biological activity of extracellular Ub (Sloper-Mould et al., 2001). In HSV-I infection model, Ub (0.087 nM) and peptides <10 kDa were capable of inducing a protective effect. Remarkably, when the concentration of Ubiquitin in Transferon was modified using commercial antibodies against Ub or by adding recombinant monomeric human Ub, the protective capacity of Transferon was diminished or augmented, respectively, which indicates that Ub is one of the active components of Transferon. Notwithstanding, considering that recombinant Ub elicited a protective effect at higher concentrations than the total Ub content of a standard batch of Transferon, there may be another peptide component that evoke a protective effect in the same model, and synergistic effects between them cannot be discarded.

Ub is a high-stable low–molecular mass protein detected in the serum of healthy humans in a concentration of less than 100 ng/ml (<10 nM), and its main source are red cells (Takada et al., 1997; Patel et al., 2006). The intracellular function of Ub is well-known. Nevertheless, the exact role of extracellular Ubiquitin is still under debate, although multiple experimental reports show that Ub or fragments of this protein attenuate pro-inflammatory responses (Jaremko et al., 2009; Majetschak, 2011). In our murine model of HSV-1 infection, a known concentration of Ub (0.087 nM) and DAMPs-like peptides (~0.4 mg/mL) is oropharyngeal deposited on five occasions during the clinical follow-up and improve the percent of survival of infected mice.

An elegant model of ischemia-reperfusion injury in C57BL/6 mice, infused monomeric Ub (1 mg/g per hour) and showed that Ub plays a protective role in myocardial remodeling post-I/R. In this model, Ub affects the cardiac function, the size area at risk/infarct, levels of serum cytokines/chemokines, and demonstrates anti-inflammatory effects, through the receptors in the vagus nerve (Scofield et al., 2019). In this way, our group previously had reported that HSV-1 infected animals treated with Transferon, at equivalent doses to that used in humans, decrease TNF-α and IL-6 and increase IFN-γ serum levels, suggesting the presence of the same anti-inflammatory phenomenon (Salinas-Jazmin et al., 2015).

**Figure 10:**
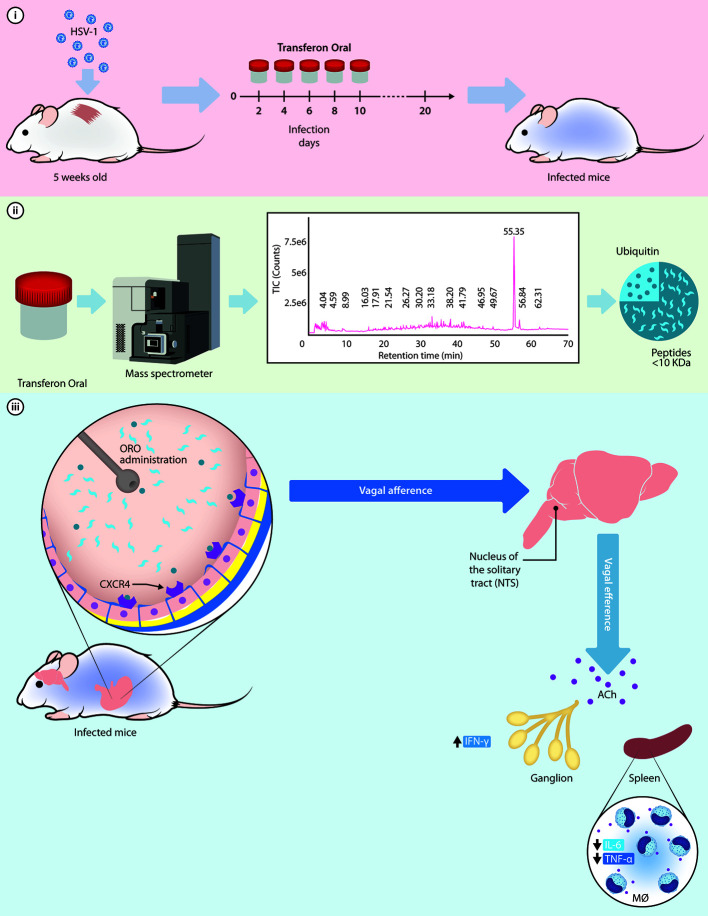
Hypothetical mechanism of action of monomeric Ub to exert its protective function in the murine model of HSV-1 infection. i) Murine model of cutaneous HSV-1 infection can be used to examine the activity of Transferon, where mice receive five doses of the treatment after infection; ii) Through a mass spectrometry analysis, the monomeric Ub and its variant Ub(-GG) were identified as the most abundant peptide component of Transferon, with a total Ub concentration of 1.003 μg/dose. iii) Once Ub, along with the rest of the peptides of Transferon, is ORO administered in the mice, it could activate the CXCR4 receptor on intragastric vagal nerve endings inducing an increase in ACh release at secondary lymphoid organs and spleen. Then, ACh attenuates the synthesis of TNF-α and IL-6, decreasing the inflammatory response in HSV-1infected mice infected and increasing the percentage of survival. HSV, Herpes simplex virus; ORO, oropharyngeal; ACh, acetylcholine; IL, interleukin; IFN, interferon; MØ, macrophages.

Considering the low concentration of the components of Transferon and the route of administration, we may hypothesize that total monomeric Ub (0.087 nM) and peptides < 10 kDa interact directly with their receptors located in the stomach, such as TLR and the CXC motif chemokine receptor 4 (CXCR4) which has been recently suggested as a putative receptor for Ub with an affinity in the medium nM range (Saini et al., 2011). CXCR4 is expressed in the afferent endings of the vagus nerve at the stomach (Hermann et al., 2008) and its activation may lead to the inhibition of the release of TNF-α by spleen macrophages through acetylcholine (ACh) signaling pathways, as proposed by **Figure 10** (Pavlov and Tracey, 2012; Bonaz et al., 2016; Browning et al., 2017; Pavlov and Tracey, 2017; Breit et al., 2018).

Our results suggest that the administration of a low concentration of a mix of monomeric Ub and peptides in <10 kDa in the stomach of animals infected with HSV-1 might activate sensory fibers that ascend in the vagus nerve to synapse in the nucleus tractus solitarius. Increased efferent signals in the vagus nerve suppress peripheral cytokine release through macrophage nicotinic receptors and the cholinergic anti-inflammatory pathway (CAIP). So that functions of extracellular Ubiquitin could constitute an evolutionarily conserved control mechanism aimed to balance the immune response and prevent exuberant inflammation induced by DAMP (Majetschak, 2011). Besides, Ub is a highly conserved across the mammalian class with extracellular anti-inflammatory effects on the innate system (Job et al., 2015), which may explain the inter-species effect of Transferon, e.g. mice (Salinas-Jazmin et al., 2015; Hernandez-Esquivel et al., 2018).

This is the first report that identify and characterize a peptide component of Transferon Oral, an hDLE extract, but further work is needed to ascertain the relevance of Ub in the HSV-1 infection murine model, its mechanism of action and its relevance among the Transferon peptide components. To challenge our hypothesis, we will perform further assays such as the determination of immunological parameters in the different treatments evaluated, such as cytokine levels and variations in lymphocyte subpopulations. We will carry out these analyzes in future work, along with the quantitation of Ub in Transferon using orthogonal analysis.

## Conclusion

The sequencing analysis by MS concluded that the main peptides of Transferon come from 22 structural proteins. One of them, the monomeric Ub, is complete or without the two-terminal Gly. The rest of the components are low–molecular mass peptides (less than <10 kDa) and are reproducible between batches. It was observed that human monomeric Ub has a protective effect and that the protective effect of Transferon correlates with the monomeric Ub content in an HSV-1 murine infection model. Taking into account these results and the reported properties of monomeric Ub, we hypothesize that Transferon down-regulate the systemic inflammatory phenomenon induced by the infection through intragastric CXCR4 receptors, favouring the recovery of infected mice; further studies are needed to demonstrate this hypothesis.

This is the first report that identifies monomeric Ubiquitin as the major peptide component of an hDLE (Transferon), based on its relative ionization in MS and that it has a similar effect to the complete extract in a murine infection model when administered by the oropharyngeal route. Further, this work evinces that Ub can be used to develop oral immunomodulatory drugs.

## Data Availability Statement

The datasets presented in this study can be found in online repositories. The names of the repository/repositories and accession number(s) can be found in the article/**Supplementary Material**.

## Author Contributions

LV-C, LP, and SP-T conceptualized the work. LF, SV-L, GM-S, ZM-P, LL-J, LV-F, and LV-C performed the assays and analyzed the results. LP and LV-C wrote the manuscript. EM-R provided critical assistance during this work. RC-S analyzed and interpreted data. All authors contributed to the article and approved the submitted version

## Funding

The mass spectrometric analysis and the *in vivo* assays were performed using the equipment and facilities of “Laboratorio Nacional para Servicios Especializados de Investigacioín, Desarrollo e Innovacioín (I + D + i) para Farmoquiicos y Biotecnológicos” (LANSEIDI-FarBiotec-CONACyT), which is part of “Unidad de Desarrollo e Investigacioín en Bioprocesos (UDIBI)-IPN”. All reagents and experimentation animals were provided by UDIBI (protocols FTU/DF/15/010-PRO, FTU/DF/16/004-PRO, FTU/P2/18/007-PRO, and UDIP20-013).

## Conflict of Interest

SP-T is involved in the development and commercialization of Transferon.

The remaining authors declare that the research was conducted in the absence of any commercial or financial relationships that could be construed as a potential conflict of interest.

The reviewer EZ declared a past co-authorship with one of the authors LP to the handling editor.
