# Supplementary material for: Sequencing Analysis and Identification of the Primary Peptide Component of the Dialyzable Leukocyte Extract “Transferon Oral”: The Starting Point to Understand Its Mechanism of Action
Source: Front Pharmacol. 2020 Oct 7;11:569039. doi: 10.3389/fphar.2020.569039 (PMC7577238; doi:10.3389/fphar.2020.569039)
Supplement: Supplementary file 1 [file DataSheet_1.pdf]

Sequencing analysis and identification of the primary peptide component of the Dialyzable Leukocyte Extract "Transferon oral": the starting point to understand its mechanism of action

Supplementary material

Luis Vallejo-Castillo[1,2,3], Liliana Favari[2], Said Vázquez-Leyva[1,3], Gabriela Mellado-Sánchez[1,3], Zaira Macías-Palacios[1,3], Leonardo E. López-Juárez[1,3], Luis Valencia-Flores[1,3], Emilio Medina-Rivero[1], Rommel Chacón-Salinas[4], Lenin Pavón[5]* and Sonia Mayra Pérez-Tapia[1,3,4]*

[1] Unidad de Desarrollo e Investigación en Bioprocesos (UDIBI), Escuela Nacional de Ciencias Biológicas, Instituto Politécnico Nacional. Mexico City, Mexico.

[2] Departamento de Farmacología, Centro de Investigación y de Estudios Avanzados del IPN. Ciudad de México, México.

[3] Laboratorio Nacional para Servicios Especializados de Investigación, Desarrollo e Innovación (I+D+i) para Farmoquímicos y Biotecnológicos (LANSEIDI-FarBiotec-CONACyT). Escuela Nacional de Ciencias Biológicas, Instituto Politécnico Nacional. Mexico City, Mexico.

[4] Departamento de Inmunología, Escuela Nacional de Ciencias Biológicas, Instituto Politécnico Nacional. Mexico City, Mexico.

[5] Laboratorio de Psicoinmunología, Dirección de Investigaciones en Neurociencias, Instituto Nacional de Psiquiatría Ramón de la Fuente. Mexico City, Mexico.

***Correspondence:**

Lenin Pavón: * Department of Psychoimmunology, National Institute of Psychiatry, “Ramón de la Fuente,” Calzada México-Xochimilco 101, Colonia San Lorenzo Huipulco, Tlalpan, 14370 Mexico City, DF, Mexico. Tel + (52) 55 41605082, Fax + (52) 55 56759980 email: lkuriaki@imp.edu.mx. Sonia Mayra Pérez Tapia: Unidad de Desarrollo e Investigación en Bioprocesos (UDIBI), Escuela Nacional de Ciencias Biológicas (ENCB) IPN. Prolongación de Carpio y Plan de Ayala S/N, Col. Casco de Santo Tomás, Del. Miguel Hidalgo, 11340. Tel +(52) 55 5729 6000 Ext. 62543. Email: sperezt@udibi.com.mx

Running title

Primary Peptide Component of the hDLE Transferon.

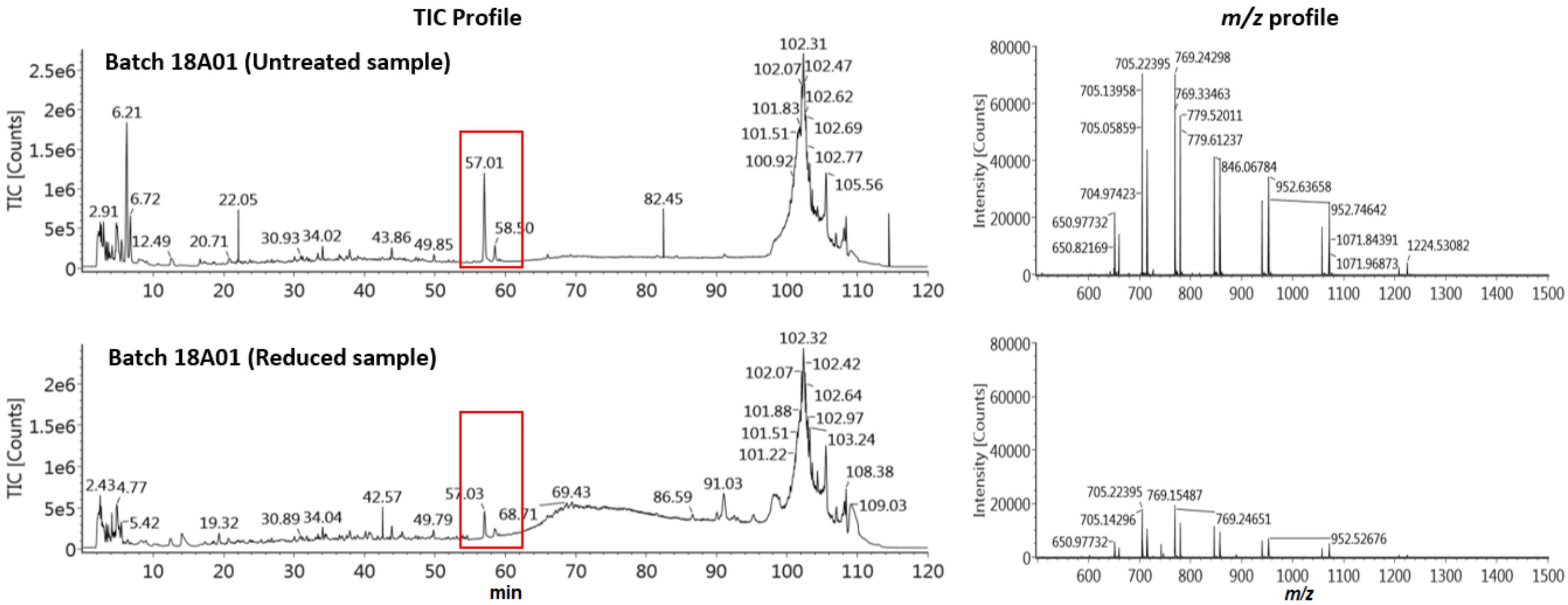

Figure S1. Identification of Ub and Ub(-GG) in reduced and non-reduced "Transferon oral" samples. A reduced "Transferon oral" sample was analyzed by MS intact analysis (1.7-µm CSH C18 column) to determine if the 57 min *m/z* signal is not associated with peptide aggregates. As observed, TIC and *m/z* signals of Ub and Ub(-GG) prevailed after chemical reduction.

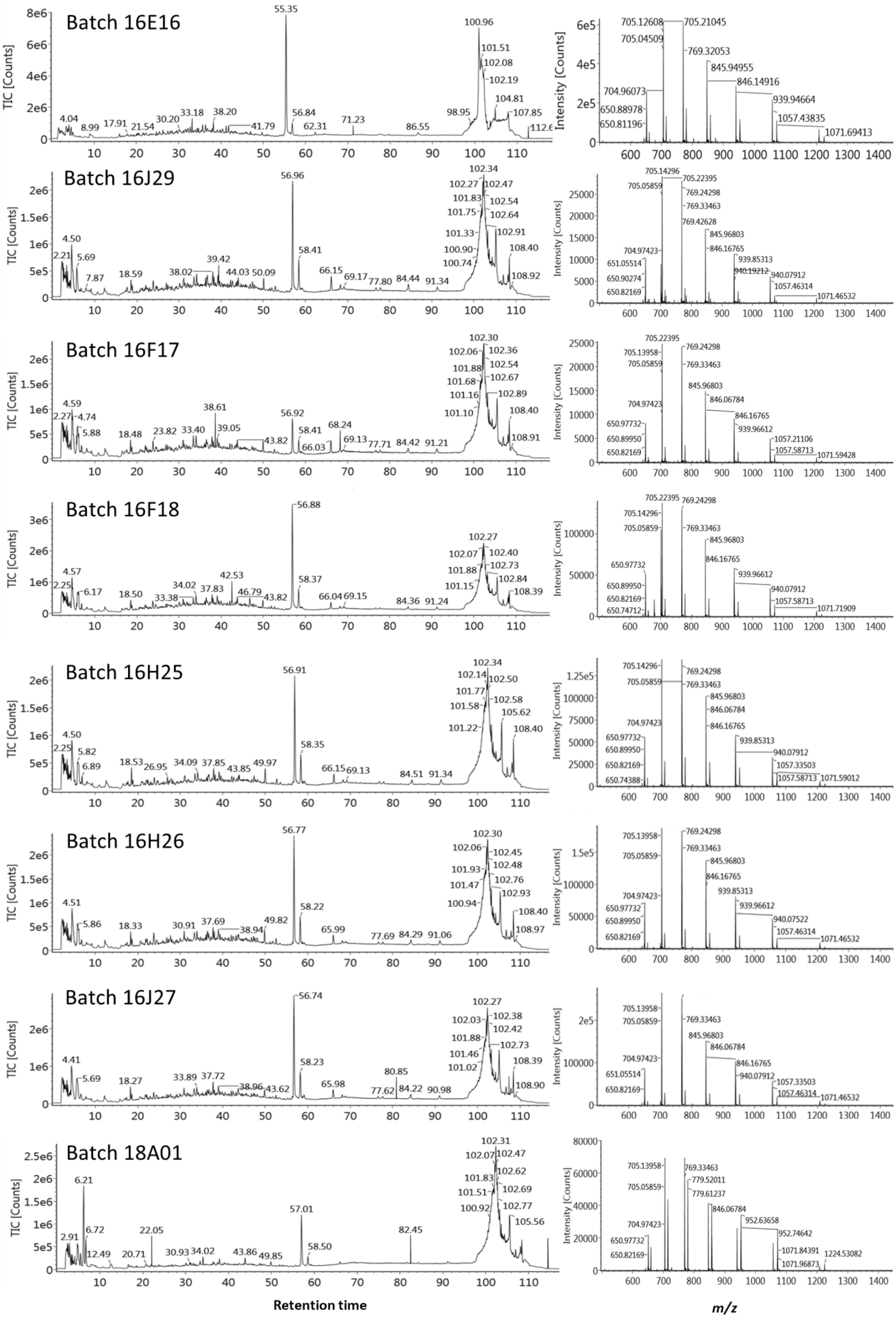

Batch 16E16
Batch 16J29
Batch 16F17
Batch 16F18
Batch 16H25
Batch 16H26
Batch 16J27
Batch 18A01
TIC [Counts]
Intensity [Counts]
Retention time
m/z

Figure S2. Identification of Ub and Ub(-GG) in different "Transferon Oral" batches. Eight batches of "Transferon Oral" were analyzed, and the *m/z* profile of the primary TIC signal (56.7 ± 0.55 min) was obtained. *m/z* patterns corresponded to Ub and Ub(-GG) in all cases. All samples were analyzed using a 1.7-µm CSH C18 column.
